# Application of learned ideal observers for estimating task-based performance bounds for computed imaging systems

**DOI:** 10.1117/1.JMI.11.2.026002

**Published:** 2024-03-20

**Authors:** Kaiyan Li, Umberto Villa, Hua Li, Mark A. Anastasio

**Affiliations:** a University of Illinois Urbana-Champaign, Department of Bioengineering, Urbana, Illinois, United States; b University of Texas at Austin, Oden Institute, Austin, Texas, United States; c Washington University School of Medicine, Department of Radiation Oncology, Saint Louis, Missouri, United States

**Keywords:** ideal observer, task-based image quality assessment, image reconstruction, deep learning

## Abstract

**Purpose:**

The performance of the ideal observer (IO) acting on imaging measurements has long been advocated as a figure-of-merit (FOM) to guide the optimization of imaging systems. For computed imaging systems, the performance of the IO acting on imaging measurements also sets an upper bound on task-performance that no image reconstruction method can transcend. As such, estimation of IO performance can provide valuable guidance when designing data-acquisition techniques by enabling the identification of designs that will not permit the reconstruction of diagnostically useful images for a specified task – no matter how advanced the reconstruction method is or plausible the reconstructed images appear. While such data space IO analyses are known conceptually, they have generally remained infeasible to widely implement. In this work, convolutional neural network (CNN) approximated IOs (CNN-IOs) are investigated for estimating the performance of data space IOs for the purpose of guiding hardware and data-acquisition designs and establishing task-based performance bounds for image reconstruction.

**Approach:**

Numerical studies that utilized a stylized breast X-ray computed tomography test bed are conducted to validate and demonstrate the approach. Signal-known-statistically and background-known-statistically (SKS/BKS) binary signal detection and discrimination tasks are addressed and the impact of the number of views and beam intensities on IO performance is investigated as a case study. The image space CNN-IO performance is also computed by use of images reconstructed by both U-Net and FBP reconstruction methods and compared to the corresponding data space CNN-IO performance to assess task-related information loss.

**Results:**

For all considered cases, task-performance bounds were established by use of the data space CNN-IO performance. A comparison of the data space and image space CNN-IO performances quantified the task-relevant information loss induced by the considered image reconstruction methods. Moreover, the U-Net reconstructed images possessed improved traditional metrics compared to those produced by the FBP method but resulted in lower image space CNN-IO performance. This demonstrates that traditional IQ measures can be misleading if task-performance is of ultimate interest.

**Conclusion:**

This work confirms that recent developments in learning-based IO approximation methods can enable the ranking of data-acquisition designs based on optimal task-performance with consideration of object variability. The work also demonstrates that such methods can enable estimation of task-based performance bounds for image reconstruction.

## 1 Introduction

It has been widely acknowledged that the use of objective measures of image quality (IQ) that quantify the ability of an observer to perform a specific task is critically important for the assessment of medical imaging systems.^1^^–^^4^ When optimizing hardware or data-acquisition designs for computed imaging systems, it is desirable to maximize the amount of task-specific information that is contained in the imaging measurements. To achieve this when signal detection tasks are considered, objective IQ measures based on the performance of the Bayesian ideal observer (IO) acting on measurement data have been advocated.^1^^–^^4^ The IO acting on such directly acquired data, as opposed to reconstructed images that represent object estimates, will be referred to as the data space IO in this work. Importantly, the performance of the data space IO represents an upper bound on task-performance that no image reconstruction method can improve upon.^5^ As such, the data space IO can also enable the assessment of task-relevant information loss induced by image reconstruction, by comparing the data space IO performance to the performance of the IO acting on reconstructed object estimates. The latter observer will be referred to as the image space IO. The ratio of these performances has been referred to as detection efficiency in the literature.^6^^–^^8^ It is also noteworthy that IO analyses of imaging systems can be interpreted in terms of information theoretic concepts.^9^^,^^10^

Data space IO analyses are now even more important than ever considering the rapid exploration of learning-based image reconstruction methods. A variety of deep learning-based image reconstruction methods are being actively developed to enable highly incomplete and noisy data acquisition designs for the purpose of minimizing data-acquisition times and/or the radiation risk to patients.^11^^–^^14^ In some cases, these learning-based methods can yield visually plausible images (i.e., object estimates) that possess encouraging image quality as measured by physical, non-task-based, metrics such as structural similarity index metric (SSIM)^15^ or peak signal-to-noise ratio (PSNR).

However, it has not been widely acknowledged in the recent literature that situations can exist in which incomplete and noisy tomographic measurement data will not permit the reconstruction of diagnostically useful images, no matter how advanced the reconstruction method is or plausible the reconstructed images appear.^16^ Estimating the performance of the data space IO provides a means for identifying these situations. Such analyses will enable the triage of data-acquisition designs and associated image reconstruction development efforts that can *never* result in a required diagnostic performance, regardless of who or what will be ultimately interpreting the images. These infeasible data-acquisition and image reconstruction method designs occur when the required diagnostic performance exceeds the performance of a data space IO.

There have been previous studies of data space IOs for signal detection tasks, but all have employed certain simplifying assumptions. For example, Sidky and Pan^8^ performed a data space IO analysis to evaluate information loss that occurs when a back-projection filtration (BPF) algorithm is employed for image reconstruction in cone-beam computed tomography (CT). There, a signal-known-exactly (SKE) and background-known-exactly (BKE) binary signal detection task was considered. Hsieh et al.^17^ also computed the data space IO for SKE/BKE binary signal detection tasks. He et al.^18^ approximated the data space IO by use of a Markov chain Monte Carlo (MCMC) method^19^ on a simple parameterized phantom. Shi et al.^20^ proposed a sub-optimal deep learning-based model observer acting on sinograms. In a different approach, Chen et al.^21^ proposed a data space IO analysis in which background variability was described by a sparsity-based image reconstruction prior. However, the capacity to perform data space IO analysis based on detection or discrimination tasks with consideration of clinically relevant object and signal variability has remained limited. This is a result of the fact that estimation of IO performance under such conditions has been generally intractable, both analytically and computationally.

There have been significant recent advances in methods for approximating the IO acting on images based on supervised learning with convolutional neural networks (CNNs).^22^^–^^25^ For example, Zhou et al.^22^ developed CNN-based methods for estimating the test statistics of IOs performing binary signal detection tasks and detection-localization tasks.^23^ More recently, Li et al.^24^^,^^25^ developed a hybrid method that involves CNNs and MCMC methods to approximate the test statistics of IOs for general detection-estimation tasks. Importantly, when implemented with appropriate stochastic models to produce training data,^26^^,^^27^ CNN-approximated IOs (CNN-IOs) can yield estimates of IO performance with consideration of realistic object and signal variability. This has provided a new capacity to conduct IO analyses of medical imaging systems.

In this work, CNN-IOs are investigated for estimating the performance of data space IOs for the purpose of guiding hardware and data-acquisition designs and establishing task-based performance bounds for image reconstruction. A stylized X-ray breast CT imaging system and an anatomically realistic stochastic object model of the breast are considered as a test bed. Data space CNN-IOs are first validated for SKE/BKE binary signal detection tasks for which analytic solutions are available. Several background-known-statistically (BKS) binary signal detection tasks and signal discrimination tasks are subsequently considered to explore the application of data space CNN-IOs for estimating performance bounds that were previously intractable. This work will advance the field of medical imaging science by paving the way for more widespread data space IO analyses of imaging technologies under clinically relevant conditions.

## 2 Background

### 2.1 Binary Signal Detection and Discrimination Tasks and the IO

A continuous-to-discrete (C-D) description of a linear imaging system^1^ is considered as

g=Hf(r)+n, (1)

where $g\in R^{N\times 1}$ is the measured image vector, f(r) denotes the object function that is dependent on the coordinate $r\in R^{k\times 1}$ with k≥2, H denotes a linear imaging operator that maps $L_{2}(R^{k})$ to $R^{N\times 1}$, and $n\in R^{N\times 1}$ denotes the measurement noise. When its spatial dependence is not important to highlight, f(r) will be denoted as f.

A binary data space signal detection task requires an observer to classify the measured image data g as satisfying either a signal-present hypothesis $H_{1}$ or a signal-absent hypothesis $H_{0}$. These two hypotheses can be described as

$$H_{0}:\;g=Hf_{b}+n=b+n,$$ (2a)

$$H_{1}:\;g=H(f_{b+s})+n=b_{s}+n,$$ (2b)

where $f_{b}$ and $f_{b+s}$ denote the signal-absent (background) and signal-present object, respectively, and $b≔Hf_{b}$ and $b_{s}≔Hf_{b+s}$ denote the measured signal-absent and signal-present image data. Similarly, a data space signal discrimination task requires an observer to choose between the hypotheses

$$H_{1}:\;g=H{(f}_{b+s_{1}})+n=b_{s_{1}}+n,$$ (3a)

$$H_{2}:\;g=H{(f}_{b+s_{2}})+n=b_{s_{2}}+n,$$ (3b)

where $f_{b+s_{1}}$ and $f_{b+s_{2}}$ denote two signal-present objects with different signals, respectively. Here, $b_{s_{1}}≔Hf_{b+s_{1}}$ and $b_{s_{2}}≔Hf_{b+s_{2}}$ denote the corresponding measured image data.

To perform these tasks, a deterministic observer computes a test statistic that maps the measured image data g to a real-valued scalar variable that is compared to a predetermined threshold τ to determine which of the two hypotheses g satisfies. By varying the threshold τ, a receiver operating characteristic (ROC) curve can be formed to quantify the trade-off between the false-positive fraction (FPF) and the true-positive fraction (TPF).^1^ The area under the ROC curve (AUC) can be subsequently calculated as a figure-of-merit (FOM) for signal detection performance.

The IO test statistic $t_{\mathrm{IO}}(g)$ is any monotonic transformation of the likelihood ratio $\Lambda_{\mathrm{LR}}(g)$. For the case of the binary detection task described in Eq. (2), $\Lambda_{\mathrm{LR}}(g)$ is defined as

$$\Lambda_{\mathrm{LR}}(g)=\frac{p(g|H_{1})}{p(g|H_{0})},$$ (4)

where $p(g|H_{1})$ and $p(g|H_{0})$ are the conditional probability density functions that describe the measured data g under the hypotheses $H_{1}$ and $H_{0}$, respectively. For the discrimination task described in Eq. (3), an analogous expression holds in terms of $H_{1}$ and $H_{2}$. When background and signal variability are considered, $\Lambda_{\mathrm{LR}}(g)$ can be rewritten as^28^

$$\Lambda_{\mathrm{LR}}(g)=\frac{\iint db\;dsp(b)p(s)p(g|b,s,H_{1})}{\int dbp(b)p(g|b,H_{0})}\equiv \iint db\;ds\Lambda_{\mathrm{SBKE}}(g|b,s)p(b|g,H_{0})p(s),$$ (5)

where $\Lambda_{\mathrm{SBKE}}(g|b,s)$ is the signal and background-known exactly (SBKE) likelihood ratio and $p(b|g,H_{0})$ is a posterior probability density function. These quantities can be computed as

$$\Lambda_{\mathrm{SBKE}}(g|b,s)=\frac{p(g|b,s,H_{1})}{p(g|b,H_{0})},$$ (6)

and

$$p(b|g,H_{0})=\frac{p(g|b,H_{0})p(b)}{\int db^{\prime }p({g|b}^{\prime },H_{0})p(b^{\prime })}.$$ (7)

To estimate $\Lambda_{\mathrm{LR}}(g)$ for this case, MCMC techniques have been proposed.^19^ However, current applications of MCMC methods have been limited to relatively simple stochastic object models (SOMs), such as a lumpy object model,^19^ a binary texture model,^29^ and a parameterized torso phantom.^18^ To circumvent this issue, supervised learning-based methods have been proposed to approximate the IO test statistic.^22^^–^^24^

### 2.2 CNN-Approximated IO

Advancements in deep learning and computing hardware have enabled new ways for estimating the IO test statistic.^22^^,^^23^^,^^30^ For use with image data, CNNs can be employed to estimate the posterior probability $p(H_{a}|g)$, which is a monotonic transform of the likelihood ratio $\Lambda_{\mathrm{LR}}(g)$.^22^ Above, $H_{a}$ denotes the alternative hypothesis $H_{1}$ for the binary detection task [Eq. (2)] and $H_{2}$ for the discrimination [Eq. (3)] task. This requires the identification of a network architecture that possesses sufficient representative capacity to enable accurate estimation of the posterior probability, and hence the IO test statistic. This can be accomplished by searching over a predetermined family of architectures.^22^^,^^23^^,^^31^ The sigmoid function is employed in the last layer of the CNN to approximate $p(H_{a}|g)$. In this way, the output of the CNN can be interpreted as probability, i.e., $p(H_{a}|g,\Theta )$. Here, Θ is the vector of the weight parameters corresponding to the CNN. The goal of training the CNN is to determine a vector Θ such that the difference between the CNN-approximated posterior probability $p(H_{a}|g,\Theta )$ and the actual posterior probability $p(H_{a}|g)$ is small.^32^ A supervised learning-based method can be employed to approximate the maximum likelihood (ML) estimate of Θ by minimizing the binary cross-entropy (BCE) loss function^22^

$$L_{\mathrm{BCE}}(\Theta )=-\overset{J}{\underset{j=1}{\sum }}\log\;p(y_{j}|g^{\left(j\right)},\Theta ),$$ (8)

where $\{(g^{\left(j\right)},y^{\left(j\right)}){\}}_{j=1}^{J}$ denote the input data $g^{\left(j\right)}$ and the corresponding label $y_{j}\in \{0,1\}$. The CNN-IO has been successfully applied to direct imaging system measurements and reconstructed images in several studies.^27^^,^^31^^,^^33^^–^^38^

## 3 Methods

Computer-simulation studies were conducted to validate and investigate the use of data space CNN-IOs to establish task-based performance bounds for image reconstruction. The established bounds were validated and assessed in a stylized simulation of X-ray breast CT. Both binary signal detection and signal discrimination tasks were considered. The impacts of the number of views and beam intensities on the established bounds were investigated.

### 3.1 Data Space CNN-IO Test Statistic Approximation

To estimate the data space IO test statistic for binary detection and discrimination tasks, CNN-IOs were trained by adopting the procedure described above.^22^ In the studies presented below, the considered two-dimensional (2D) imaging system measures data that are described by two coordinates. Therefore, the input to the data space CNN-IO was the image data g, arranged as a 2D matrix. In the data space CNN-IOs, each convolutional layer in the CNN comprised 64 filters with 5×5 spatial support followed by a Leaky ReLU activation function. A max-pooling layer following the last convolutional layer was employed to sub-sample the feature maps. A final fully connected (FC) layer with a sigmoid activation function was employed. The BCE loss function was considered and the CNN was optimized to estimate the posterior probability $p(H_{a}|g,\Theta )$, which is a monotonic transformation of the likelihood ratio $\Lambda_{\mathrm{LR}}(g)$.

For determining an effective data space CNN-IO architecture, the training process started from a CNN architecture with one convolutional layer and gradually added more layers. This training process was stopped when adding an additional layer decreased the cross-entropy by <1.0% on the validation dataset. The CNN having the minimum validation cross-entropy was selected as the data space CNN-IO in the explored architecture family. Additional training details and a description of the employed datasets are provided in Sec. 3.8.

### 3.2 Stylized X-Ray Breast CT Imaging Systems

In this study, simulated projection data corresponding to a canonical fan-beam CT imager with a linear detector geometry was employed. To produce these data, the C-D forward operator was approximated by a discrete-to-discrete operator that was implemented by use of the *Radon-torch* toolbox.^39^ The scanning angular range of the modeled fan-beam system was 360 deg and different numbers of evenly spaced tomographic views were considered. The assumed distance between the X-ray source and the center of the object, and the distance between the detector and the center of the object were 400 and 400 mm, respectively. The number of detector elements was 512, and each element was 0.8 mm in size.

Noisy projection data g were generated as^1^^,^^39^

g^=T−1{Poi[T(Hf)]}, (9)

where Poi(·) is a Poisson noise generator acting the transformed measurement data T(Hf). Here, $T(x)=I_{0}\;\exp(-x)$, and $T^{-1}(x)=\log[\frac{I_{0}}{x}]$, where $I_{0}$ is the beam intensity.

### 3.3 Stochastic Object and Lesion Models

The stochastic object model (SOM) developed under the US Food and Drug Administration’s (FDA) Virtual Imaging Clinical Trials for Regulatory Evaluation (VICTRE) project^40^ was employed to create an ensemble of to-be-imaged 2D objects that represented slices through a stochastic numerical breast phantom. The VICTRE SOM is inherently three-dimensional (3D) but a 2D SOM was formed by extracting 2D slices from the produced 3D breast phantoms. The dimension of these slices was 368×368  pixels with a pixel size of 0.4 mm. The X-ray energy was assumed to be 30 keV^41^ and the linear attenuation coefficient values (μ) in unit of ${\mathrm{cm}}^{-1}$ at this energy were assigned to the voxels corresponding to each of the 10 tissue types in the generated numerical breast phantoms.^42^

**Fig. 1:**
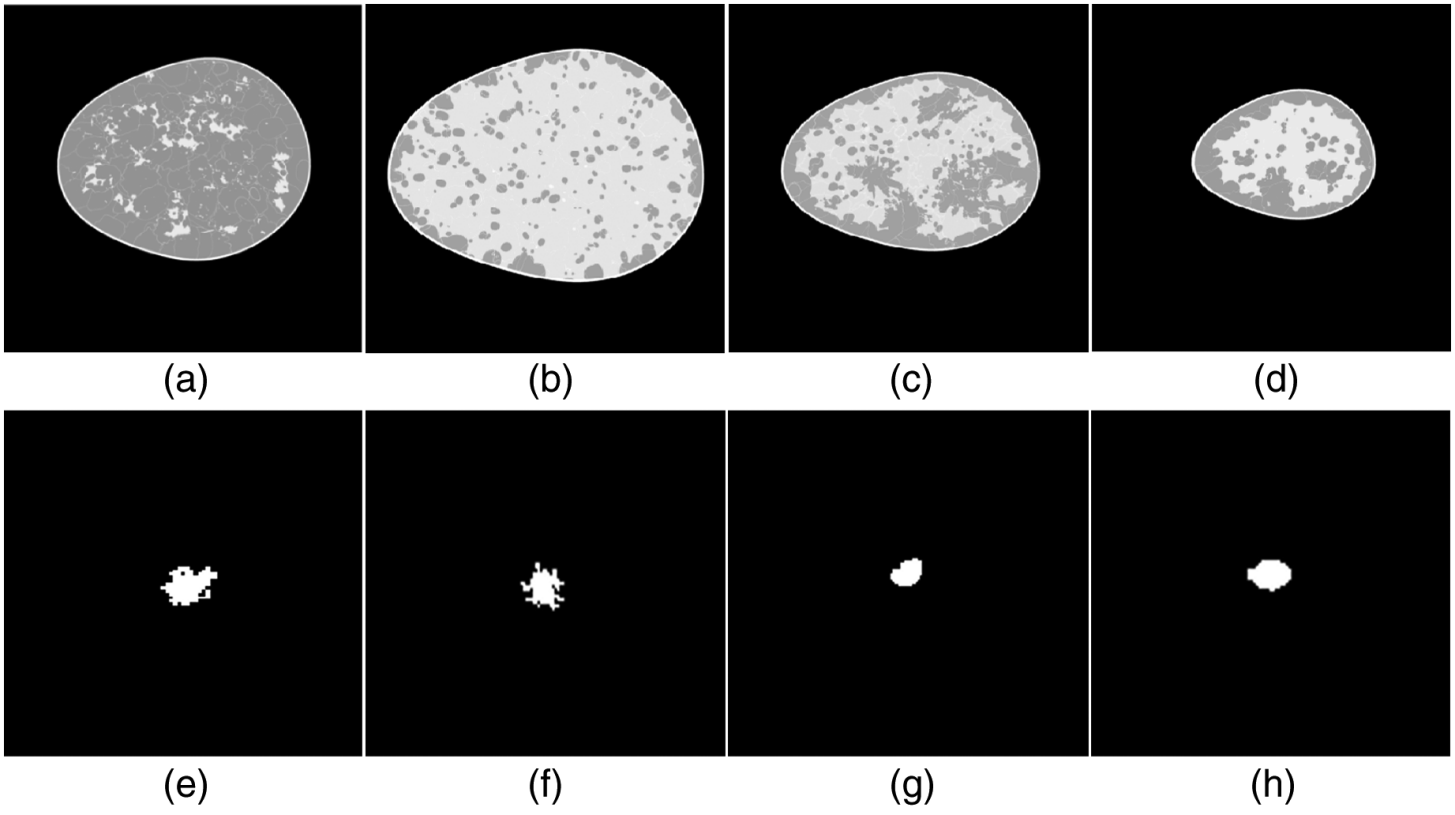
Realizations of (a)–(d) the employed backgrounds produced via the VICTRE SOM; (e) and (f) spiculated stochastic lesion; (g) and (h) smooth stochastic lesion. The realizations of stochastic lesions are enlarged by 400% to enable better visualization.

The VICTRE stochastic lesion model^43^ was employed to create ensembles of to-be-detected signals. The stochastic lesion model described central 2D slices of 3D mass lesions of diameter 5 mm. Both spiculated and smooth mass lesions were considered and a plausible value of μ corresponding to 30 keV was assigned based on the literature.^44^ To create signal present (SP) objects, realizations of the stochastic lesion were inserted into background realizations produced by use of the VICTRE SOM by replacing the μ in the background object with those of the lesions. Figure 1 shows realizations of employed backgrounds produced by use of the VICTRE SOM (top row) and realizations of the stochastic lesion models for both spiculated and smooth mass lesions (bottom row).

### 3.4 SKE/BKE Validation Study

Signal-known-exactly (SKE) and background-known-exactly (BKE) binary signal detection tasks were employed to validate the data space CNN-IO method. For this purpose, two SKE/BKE tasks were chosen for which the data space IO test statistic could be analytically computed. In one task the measurement noise model was considered to be pure Poisson and in the second it was specified as independent and identically distributed (iid) Gaussian. In the considered data-acquisition design, a total of 256 views were employed that were evenly spaced over 360 deg. The deterministic background and signal for the SKE/BKE tasks were specified as realizations of the VICTRE SOM [Fig. 1(a)] and spiculated stochastic lesion model [Fig. 1(e)], respectively. For the task with Poisson noise, the measurement noise was generated according to Eq. (9), where $I_{0}=e^{15}$. For the case of Gaussian noise, iid Gaussian noise with a standard deviation of 0.6 was employed.

### 3.5 Investigation of Performance Bounds for Varying Numbers of Tomographic Views

Both binary signal detection tasks and signal discrimination tasks were considered and task-based performance bounds were established by estimating the data space CNN-IO performance. A total of 256, 128, 64, and 32 tomographic views were considered that were evenly spaced over 360 deg. The beam intensity was fixed, and both the Poisson and Gaussian noise models described above were employed.

To assess task-related information loss induced by image reconstruction, the CNN-IO performance on reconstructed object estimates was also estimated. Hereafter, this observer will be referred to as an *image space* CNN-IO. Both U-Net^12^^,^^13^ and conventional filtered back-projection (FBP) reconstruction algorithm with a Ram-Lak filter^1^ were considered. The image space and data space CNN-IO performances, as measured by ROC curves and AUC values, were then compared. The details of the designed studies are described below.

#### 3.5.1 Studies involving binary signal detection tasks

The following three BKS binary signal detection tasks of varying difficulty were considered

• **Task 1:** SKE/BKS binary signal detection task;
• **Task 2:** Signal-known-statistically (SKS) and BKS binary signal detection task with fixed signal location;
• **Task 3:** SKS/BKS binary signal detection task with random signal location.

For Task 1, a realization of the spiculated stochastic lesion [Fig. 1(e)] was considered as the deterministic signal with $\mu =0.404\;{\mathrm{cm}}^{-1}$. The VICTRE SOM was employed to describe the random background. For task 2, each signal was randomly selected from a library of 10,000 realizations of the stochastic lesion. For each signal realization, the corresponding μ was sampled from a Gaussian distribution $\mu ∼N(0.404,0.026^{2})$, in units of ${\mathrm{cm}}^{-1}$.^44^ For task 3, the signal was randomly selected from the library and its μ value was also randomly sampled as in task 2. In addition, the signal was randomly located within potential locations provided by the SOM following a discrete uniform distribution. Poisson noise was added to the projections with $I_{0}=e^{15}$ in Eq. (9).

#### 3.5.2 Studies involving signal discrimination tasks

In addition to binary signal detection tasks, signal discrimination tasks were considered, where the data space CNN-IO decided whether a spiculated mass or a smooth mass is present. In this SKE/BKS signal discrimination task, a pair of realizations of both spiculated and smooth stochastic lesions were employed as the deterministic to-be-discriminated signals, as shown in Figs. 1(e) and 1(g). The VICTRE SOM was employed to describe the random background. Poisson noise was added to the projections with $I_{0}=e^{16}$ according to Eq. (9).

### 3.6 Investigation of Performance Bounds for Varying Incident Beam Intensities

The impact of the beam intensity on task-based performance bounds was investigated. The value of $I_{0}$ in Eq. (9) was gradually reduced and the corresponding impact on the established bounds was quantified. Poisson noise was added to the projections and the values $I_{0}=\{e^{17},e^{16},e^{15},e^{14}\}$ were considered to simulate different beam intensities. A total of 128 views were employed that were evenly spaced over 360 deg. The three BKS binary signal detection tasks described in Sec. 3.5.1 were considered in this study. Task-based performance bounds were estimated by computing the data space CNN-IO performance on the noisy tomographic measurements. Task-related information loss induced by image reconstruction for the different cases was assessed as described in Sec. 3.5. Specifically, the image space CNN-IO performance was computed by use of images reconstructed by both the U-Net and FBP reconstruction methods and compared to the corresponding data space CNN-IO performance.

### 3.7 System Ranking Study

An imaging system ranking study was considered to demonstrate the impact of object variability when establishing task-based performance bounds. Two different imaging systems with the same dose budget^45^ were considered. For the first imaging system, “system 1,” a total of 256 tomographic views were evenly distributed over 360 deg, and $I_{0}=e^{15}$ in Eq. (9) was considered. For the second imaging system, “system 2,” a total of 32 tomographic views and $I_{0}=8e^{15}$ in Eq. (9) were considered. The two imaging systems were ranked by use of the estimated data space CNN-IO performance for the binary signal detection tasks described as follows.^23^^,^^46^

**Fig. 2:**
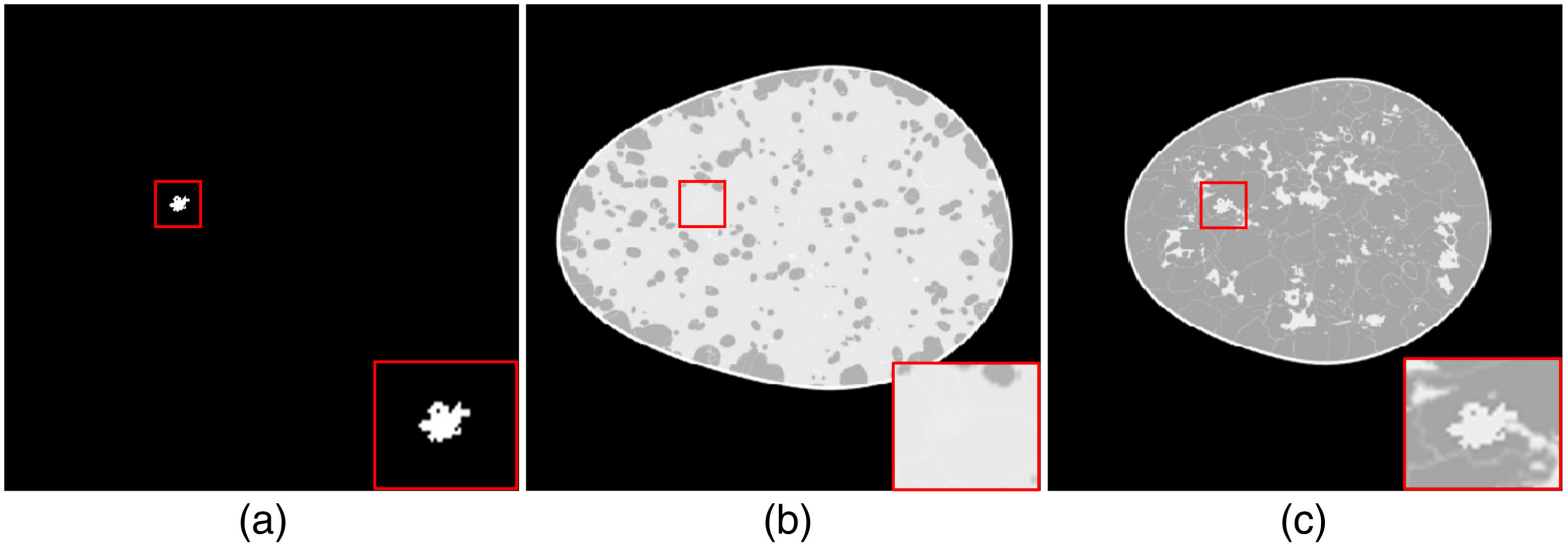
Examples of (a) the employed spiculated lesion and SP objects for the (b) BKE 1 and (c) BKE 2 binary signal detection tasks in the system ranking study. The red box indicates the signal region. The lesion contrast for the (b) BKE 1 task was relatively low and was relatively high for the (c) BKE 2 task.

Two SKE/BKE tasks and a SKE/BKS task were employed. For the first SKE/BKE task, referred to as “BKE 1,” a spiculated lesion with μ=0.383 was inserted into a selected “dense” background object where the μ of the lesion was close to that of the background around the signal (i.e., lower signal contrast). For the second SKE/BKE task, referred to as “BKE 2,” the same lesion was inserted into another selected “fatty” background object that resulted in higher signal contrast. Examples of the employed spiculated lesion and SP objects for the two SKE/BKE tasks are shown in Fig. 2.

For the SKE/BKS task, the same lesion was employed and the VICTRE SOM was employed to describe the random background. The IO performance was computed analytically for the SKE/BKE tasks.^1^ The data space CNN-IO was employed to estimate IO performance for the SKE/BKS tasks.

### 3.8 CNN-IO Training Details and Datasets

The standard convention of utilizing separate training/validation/testing datasets was adopted. For training the data space CNN-IO for SKE/BKE detection tasks, each mini-batch contained 500 pairs of fixed signal-present and signal-absent measurements. The measurement noise was generated on-the-fly and added to noiseless mini-batches.^22^ For training the data space CNN-IO for the BKS tasks, 114,400 background objects were generated. A “semi-online learning” method^22^ was employed to mitigate overfitting that can be caused by insufficient training data. At each iteration of the training process, a mini-batch consisting of 100 background objects was drawn from the generated background object dataset. For binary signal detection tasks, signals were inserted into half of the drawn background objects to create signal-present objects. For signal discrimination tasks, spiculated and smooth signals were inserted into each half of the drawn background objects. The fan-beam forward operator described in Sec. 3.2 was applied to the mini-batch and measurement noise was added subsequently to generate noisy measurement data.

For estimating the image space CNN-IO performance on the U-Net and FBP reconstructed images, the corresponding reconstruction operator (pre-trained U-Net and FBP) was applied to the generated noisy measurements. The reconstructed images were then employed as inputs for image space CNN-IO model training and testing. The Adam optimizer^47^ with a learning rate of 0.0001 was employed for both data space and image space CNN-IO training.

For all considered tasks, the validation dataset included 2000 pairs of signal-present and signal-absent raw measurements. Finally, the testing dataset comprised 10,000 signal-present images and 10,000 signal-absent raw measurements.

### 3.9 Evaluation Metrics

ROC analysis was conducted and area under the curve (AUC) values were computed and employed to quantify the data space and image space CNN-IO performance. The ROC curves were fit by use of the Metz-ROC software^48^ that employs the proper binormal model.^49^ The uncertainty of the AUC values was estimated as well. For comparison, two commonly used physical metrics, PSNR and SSIM, were employed as task-agnostic measures to assess the images reconstructed by U-Net-based methods and the FBP algorithm.

## 4 Results

### 4.1 SKE/BKE Validation Study

**Fig. 3:**
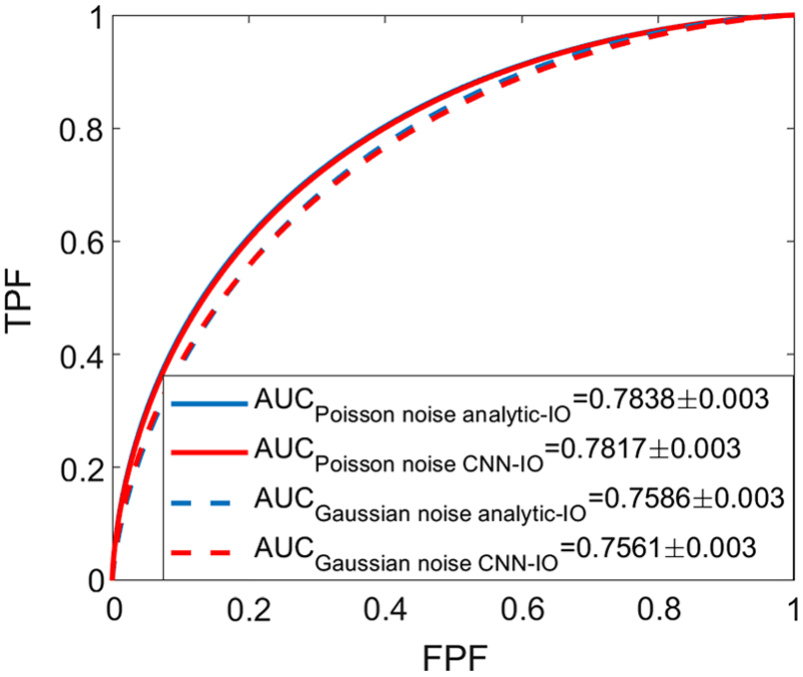
For both SKE/BKE cases with Poisson (solid curves) and Gaussian (dashed curves) noise, the ROC curves produced by the analytical computation (blue curves) and the CNN-IO (red curves) were statistically equivalent.

Figure 3 shows the ROC curves produced by the data space CNN-IO (red curves) and analytical computation (blue curves) for the SKE/BKE cases with both Poisson (solid curves) and Gaussian (dashed curves) noise. For both cases, the AUC values produced by the data space CNN-IO were statistically equivalent to those computed analytically.

### 4.2 Task-Performance versus Number of Tomographic Views

#### 4.2.1 Binary signal detection tasks

**Fig. 4:**
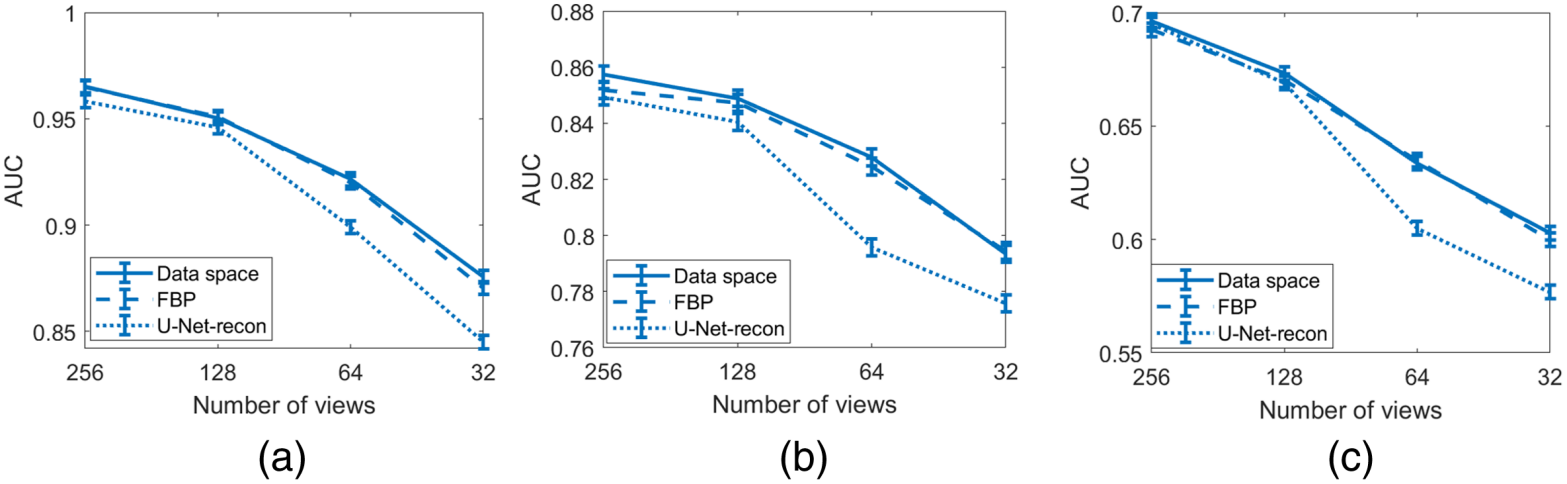
The relationships between AUC and the number of views were quantified. The binary signal detection tasks defined in Sec. 3.5.1 were considered and the results here correspond to (a) task 1, (b) task 2, and (c) task 3. The CNN-IO performance on raw tomographic measurements (solid), FBP reconstructed images (dashed), and U-Net reconstructed images (dotted) was estimated.

Figure 4 shows the estimated task-based performance bounds for different numbers of views (256, 128, 64, and 32) for the three considered tasks. As expected, for all cases, it was observed that the established bounds decreased as a function of the number of views. Moreover, a comparison of the data space and image space CNN-IO performances revealed that the amount of task-relevant information loss induced by the considered image reconstruction methods increased when the number of tomographic views was reduced.

**Fig. 5:**
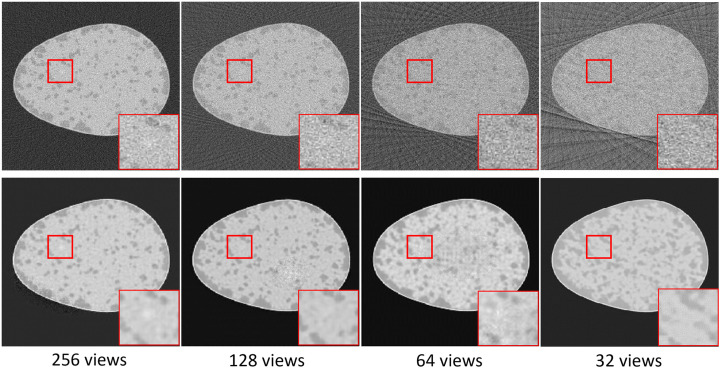
Examples of the signal-present reconstructed images from 256, 128, 64, and 32 views, respectively. The images were reconstructed by use of the FBP algorithm (upper row) and the U-Net-based method (bottom row). The red box contains the signal.

As shown in Fig. 5 and Table 1, the U-net-based method greatly improved traditional IQ measures and visual appearances but not task-based IQ measures when compared with the FBP method for all considered numbers of views. This is consistent with the fact that traditional IQ measures may not correlate with objective measures of IQ.

**Table 1:** The relationships between traditional measures (PSNR and SSIM) and the number of views were quantified. Both the U-Net-based and FBP methods were applied to the datasets used in the binary signal detection tasks described in Sec. 3.5.1. The U-Net-based methods greatly improved traditional IQ measures but not task-based IQ measures as compared to the FBP method.

Number of views		256	128	64	32
(a) Traditional measures when the dataset used in **Task 1** was employed					
PSNR	**Task1**: FBP	46.8970	44.7962	42.9541	41.8944
	**Task1**: U-Net-recon	65.8065	63.6988	63.0880	62.2104
SSIM	**Task1**: FBP	0.7879	0.7685	0.7268	0.6861
	**Task1**: U-Net-recon	0.9997	0.9994	0.9991	0.9988
AUC	**Task1**: FBP	0.9648	0.9510	0.9201	0.8703
	**Task1**: U-Net-recon	0.9583	0.9461	0.8990	0.8450
(b) Traditional measures when the dataset used in **Task 2** was employed.					
PSNR	**Task2**: FBP	46.8476	44.7739	42.9288	41.8465
	**Task2**: U-Net-recon	65.7915	63.6836	63.0725	62.2081
SSIM	**Task2**: FBP	0.7879	0.7685	0.7267	0.6860
	**Task2**: U-Net-recon	0.9997	0.9994	0.9991	0.9987
AUC	**Task2**: FBP	0.8518	0.8473	0.8246	0.7945
	**Task2**: U-Net-recon	0.8494	0.8404	0.7957	0.7758
(c) Traditional measures when the dataset used in **Task 3** was employed.					
PSNR	**Task3**: FBP	46.8253	44.7572	42.8937	41.7809
	**Task3**: U-Net-recon	65.7865	63.6799	63.0697	62.2035
SSIM	**Task3**: FBP	0.7879	0.7684	0.7267	0.6859
	**Task3**: U-Net-recon	0.9997	0.9994	0.9990	0.9985
AUC	**Task3**: FBP	0.6925	0.6701	0.6349	0.5998
	**Task3**: U-Net-recon	0.6950	0.6689	0.6049	0.5768

#### 4.2.2 Signal discrimination tasks

**Fig. 6:**
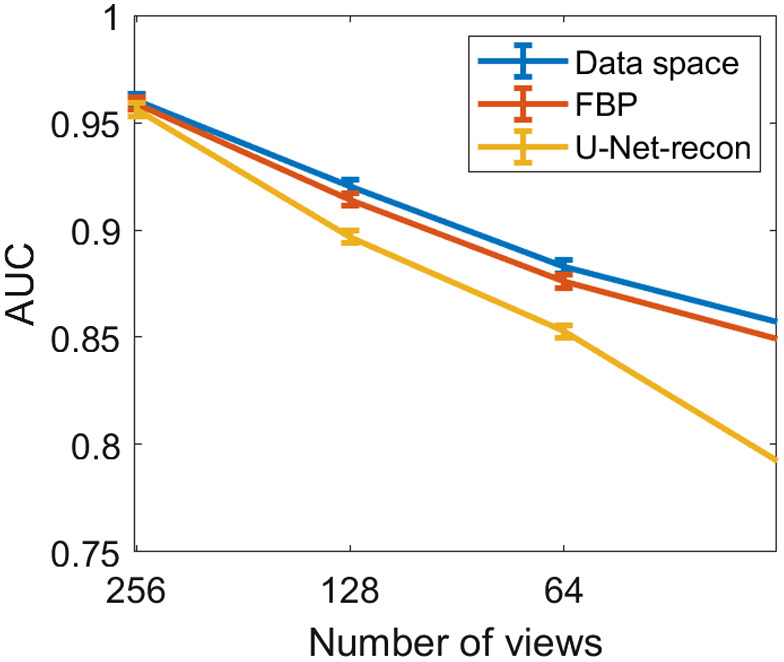
The relationships between AUC and the number of views were quantified. Signal discrimination tasks were considered. The IO performance on raw tomographic measurements (blue), FBP reconstructed images (red), and U-Net reconstructed images (yellow) were estimated.

Similar results were observed for the signal discrimination tasks. Figure 6 shows the estimated task-based performance bounds for different numbers of views. It was observed that the performance of the image space CNN-IO on the U-Net reconstructed images decreased faster as a function of the number of views as compared to the case where the FBP method was employed. Hence, relative to the data space CNN-IO, the U-Net-based method increased the amount of task-related information loss.

Despite this, the U-Net-based methods improved the subjective visual appearance and physical measures of IQ compared to the FBP method, as demonstrated in Fig. 5 and Table 2.

**Table 2:** The relationships between traditional measures (PSNR and SSIM) and the number of views were quantified. Both the U-Net-based and FBP methods were applied to the datasets used in the signal discrimination task described in Sec. 3.5.2. The U-Net-based methods greatly improved traditional IQ measures but not task-based IQ measures as compared to the FBP method.

Number of views		256	128	64	32
PSNR	FBP	51.8970	47.7969	44.3547	43.8965
	U-Net-recon	66.8397	66.2255	65.7787	64.6567
SSIM	FBP	0.8679	0.8274	0.7770	0.7459
	U-Net-recon	0.9997	0.9996	0.9995	0.9995
AUC	FBP	0.9588	0.9142	0.8760	0.8492
	U-Net-recon	0.9561	0.8968	0.8527	0.7921

### 4.3 Task-Performance versus Beam Intensities

**Fig. 7:**
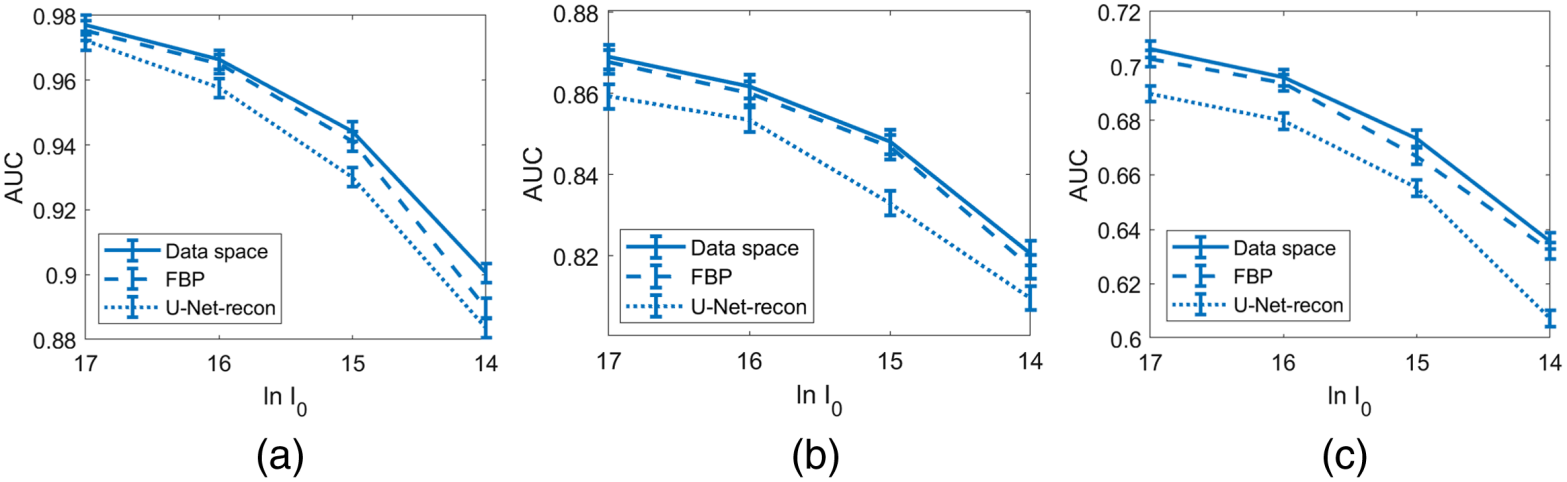
The relationships between AUC and $I_{0}$ were quantified. The binary signal detection tasks defined in Sec. 3.5.1 were considered and the results here correspond to (a) task 1, (b) task 2, and (c) task 3. The IO performance on raw tomographic measurements (solid), FBP reconstructed images (dashed), and U-Net reconstructed images (dotted) were estimated.

**Fig. 8:**
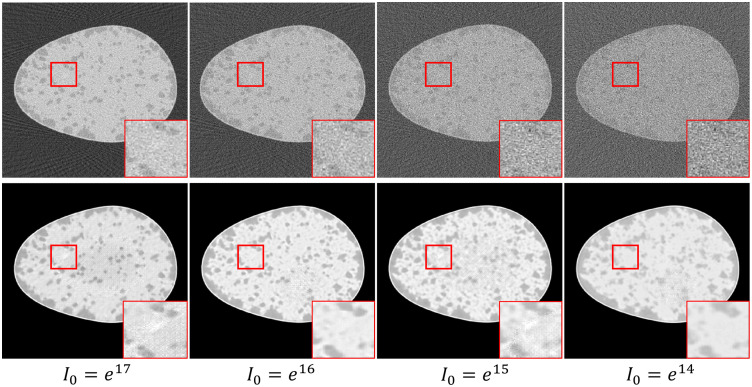
Examples of the signal-present reconstructed images from the simulated imaging systems with $I_{0}=\{e^{17},e^{16},e^{15},e^{14}\}$, respectively. The images were reconstructed by the FBP algorithm (upper row) and the U-Net-based methods (bottom row). The red box contains the signal.

The estimated task-based performance bounds for when varying beam intensities $I_{0}$ were considered are shown in Fig. 7. As expected, it was observed that the estimated bounds corresponding to the data space CNN-IO performance decreased as a function of $I_{0}$. Again, as shown in Fig. 8 and Table 3, the U-Net reconstructed images possess improved physical metrics compared to those produced by the FBP method but resulted in lower image space CNN-IO performance.

**Table 3:** The relationships between traditional measures (PSNR and SSIM) and $I_{0}$ were quantified. Both the U-Net-based and FBP methods were applied to the datasets used in the binary signal detection tasks described in Sec. 3.5.1. The U-Net-based methods greatly improved traditional IQ measures but not task-based IQ measures as compared to the FBP method.

$I_{0}$		exp(17)	exp(16)	exp(15)	exp(14)
(a) Traditional measures when the dataset used in **Task 1** was employed.					
PSNR	**Task1**: FBP	48.3906	46.9774	44.8291	42.4748
	**Task1**: U-Net-recon	65.9381	63.9233	63.3843	62.7699
SSIM	**Task1**: FBP	0.8694	0.8271	0.7691	0.7023
	**Task1**: U-Net-recon	0.9998	0.9995	0.9993	0.9990
AUC	**Task1**: FBP	0.9753	0.9650	0.9411	0.8897
	**Task1**: U-Net-recon	0.9722	0.9576	0.9301	0.8835
(b) Traditional measures when the dataset used in **Task 2** was employed.					
PSNR	**Task2**: FBP	48.3748	46.7823	44.7712	42.3087
	**Task2**: U-Net-recon	65.9288	63.9342	63.3783	62.7681
SSIM	**Task2**: FBP	0.8694	0.8271	0.7691	0.7023
	**Task2**: U-Net-recon	0.9998	0.9995	0.9993	0.9989
AUC	**Task2**: FBP	0.8678	0.8601	0.8468	0.8173
	**Task2**: U-Net-recon	0.8593	0.8535	0.8330	0.8096
(c) Traditional measures when the dataset used in **Task 3** was employed.					
PSNR	**Task3**: FBP	48.3627	46.7725	44.7572	42.2035
	**Task3**: U-Net-recon	65.9175	63.9212	63.3679	62.7674
SSIM	**Task3**: FBP	0.8694	0.8271	0.7690	0.7022
	**Task3**: U-Net-recon	0.9998	0.9995	0.9992	0.9988
AUC	**Task3**: FBP	0.7027	0.6938	0.6669	0.6322
	**Task3**: U-Net-recon	0.6898	0.6798	0.6552	0.6074

### 4.4 System Ranking Test Case

**Fig. 9:**
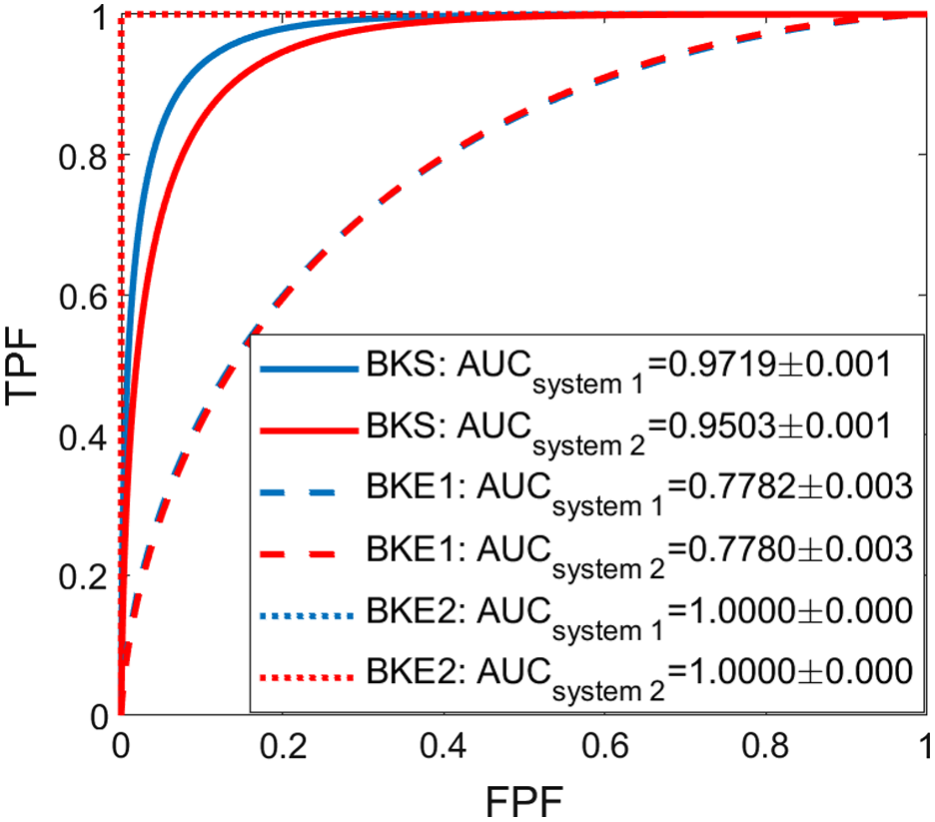
The ROC curves correspond to the data space IOs for the SKE/BKS (solid), BKE 1 (dashed), and BKE 2 (dotted) binary signal detection tasks. Both “system 1” (blue) and “system 2” (red) were considered in this test case. The rankings of the two imaging systems were different when object variability was and was not considered. The two imaging systems could not be distinguished when the BKE tasks were considered.

As shown in Fig. 9, the rankings of the two imaging systems produced by data space IOs were different when object variability was and was not considered. When object variability was considered (BKS, solid lines), “system 1” > “system 2,” whereas when object variability was not considered (BKE1/BKE2, dashed/dotted lines), “system 1” ≈ “system 2.” In addition, it was observed that the choice of background greatly impacted the established task-based performance bounds for the BKE task. For a “dense” object (BKE1, dashed lines) in Fig. 9, the task-based performance bounds were relatively low for both imaging systems due to the low signal contrast. For a “fatty” object (BKE2, dotted lines) in Fig. 9, the established task-based performance bounds were high with AUC=1 for both two imaging systems. These observations are consistent with the well-known fact that consideration of object variability is critical when computing objective measures of IQ.^1^

## 5 Summary and Discussion

Data space CNN-IOs were investigated for estimating the performance of IOs acting on tomographic measurement data for the purpose of establishing task-based performance bounds for image reconstruction when clinically relevant object variability was considered. A stylized simulation of X-ray breast CT was employed as an example. Both binary signal detection tasks and signal discrimination tasks were considered to study the impacts of the number of views, and beam intensity on task-based performance bounds for image reconstruction. Both U-Net-based methods and conventional FBP algorithms were employed as examples of image reconstruction methods in this paper. It should be noted that the considered imaging systems, tasks, and reconstruction methods were only examples to demonstrate the feasibility of the proposed methodology. The proposed methodology can be repeated for situations where different imaging systems, reconstruction methods, and tasks are considered. This represents the primary impact of the work on the field of medical image science.

The performance bounds estimated by use of data space CNN-IOs can be employed to identify situations in which the reconstruction of images cannot enable a specified diagnostic performance, independent of the image reader. This is a timely capability, because deep learning methods are being actively developed for image reconstruction from degraded and incomplete measurements but are not routinely evaluated by use of objective IQ measures. The ability of such methods to produce plausible images that possess encouraging traditional IQ measures does not imply that the images will be diagnostically useful. The presented methodology may enable the more efficient development and exploration of such image reconstruction methods for medical imaging applications.

The data space CNN-IO methodology currently processes certain limitations. Being a data-driven IO approximation method, the CNN-IO requires a large amount of training data to accurately approximate the IO performance. This can potentially be achieved when virtual imaging studies^50^^,^^51^ are performed and a relevant SOM is employed to produce an ensemble of to-be-imaged objects. A challenge in estimating the data space IO performance by use of CNNs is the specification of the collection of model architectures to be systematically explored. In this study, we manually explored a family of CNNs that possess different numbers of convolutional layers. By adding more layers, the representation capacity of the network was increased and the test statistic could be more accurately approximated. However, this method is heuristic and leaves certain parameters like the size of convolutional filters unoptimized. Recent works in network architecture search (NAS)^52^ provide methods that optimize the network architecture automatically in the training process. This may represent a more advanced approach to jointly optimizing the network architecture and weights to approximate the data space IO.

There remain numerous important topics for future investigation. In this work, the CNN-IO was directly applied to raw tomographic measurements. The benefit of introducing a “physical layer” when approximating the data space CNN-IO should be further investigated, considering the difference in data representation between data space and image space. A “physical layer” can be interpreted as a transform from the measurement to the object domain that preserves task-relevant information, e.g., a pseudo-inverse operation. Another interesting topic for future studies is the adoption of a recently proposed sampling-based IO approximation method^53^ for estimating the data-space IO. Investigating the potential benefits of this advanced technique as compared to existing supervised learning-based methods for data space IO analyses has not been explored. Furthermore, it will be important to extend the proposed data space IO method to 3D cone-beam CT, although certain challenges must be addressed. A primary challenge arises from the need for 3D network architectures to accurately approximate the IO in this scenario, which will require increased computational resources for training as compared to 2D CNNs. Future research should additionally investigate the data space CNN-IO methodology to other imaging problems with consideration of more complicated tasks such as detection-localization^23^ and detection-estimation tasks.^24^

## 6 Appendix A: U-Net-based Reconstruction Method

The U-Net-based method was applied to the image domain to reduce image artifacts as a post-processing technique and employed filtered back projection (FBP) reconstructed images as input data. The Ram-Lak filter was employed for the FBP algorithm. Given an FBP reconstructed image $f_{\mathrm{FBP}}$, the U-Net-based method can be described generically as

$$f_{\mathrm{recon}}=F(f_{\mathrm{FBP}},\Theta ),$$ (10)

where the mapping F denotes the U-Net network that is parameterized by the weight vector Θ and $f_{\mathrm{recon}}$ denotes the U-Net reconstruction estimate. In this paper, the true object f in Eq. (9) was employed as the target image and $f_{\mathrm{recon}}$ can be interpreted as an estimate of f.

The architecture of the employed U-Net is described below. Specifically, a U-Net consists of multiple stages with different spatial dimensions connected by pooling layers in the first half and up-convolutional layers in the second half. After each pooling operation, the spatial dimension was halved while the number of channels for each convolution layer was doubled. For the up-convolution operation, the spatial dimension was doubled while the number of channels for each convolution layer was halved. At each resolution level, two convolutional layers with 32 convolutional filters of dimension 3×3 were employed. Each convolutional layer was followed by a ReLU activation function and batch normalization (BN). A concatenation operation was also employed for each resolution level to incorporate the higher-resolution structural information into each up-convolution operation. At the final layer, a 1×1 convolutional layer was employed to formulate the reconstruction estimate. This multiscale network enhances the receptive field and may better suppress both local and global artifacts.

Mean square error (MSE) that measures the $L^{2}$ distance between the reconstructions and target images was employed as the loss function to optimize the U-Net in this study. For all considered tasks, a training dataset containing 57,200 signal-present and 57,200 signal-absent noisy FBP reconstructed images was employed. The validation dataset included 2000 pairs of signal-present and signal-absent noisy images. Finally, the testing dataset comprised 10,000 signal-present images and 10,000 signal-absent noisy FBP reconstructed images. The Adam optimizer^47^ with a learning rate of 0.0001 was employed for training.

## 7 Appendix B: The Specification of Optimal CNN Architecture to Approximate the IO

**Fig. 10:**
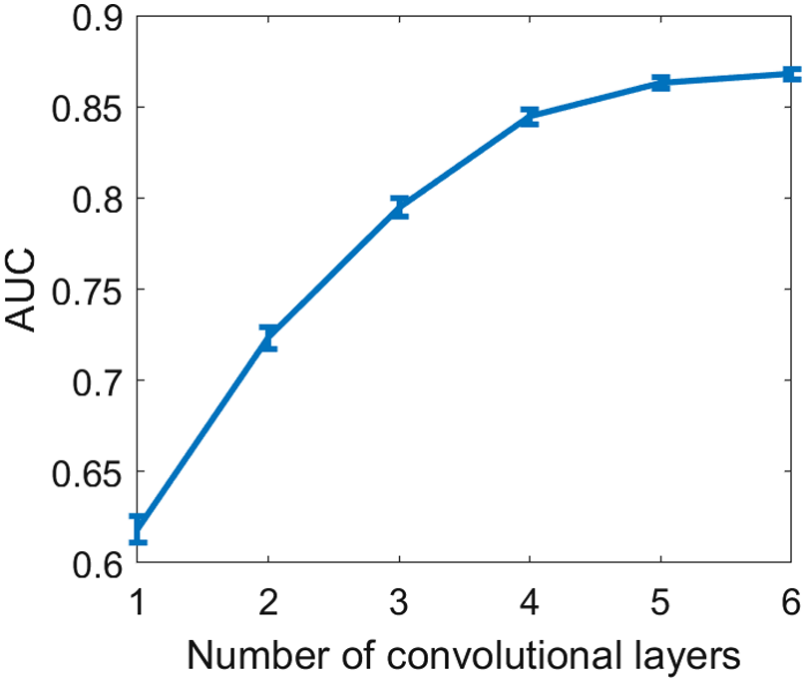
The training process of the CNN-IO was demonstrated. **Task 2** defined in Sec. 3.5.1 was considered as an example. The AUC values were not significantly increased after six convolutional layers were included.

The optimal architecture of CNNs to approximate the IO was explored and the impact of the number of convolutional layers on the binary signal detection task performance was investigated. **Task 2** defined in Sec. 3.5.1 was considered as an example. The training process of CNN-IO started from a CNN architecture with one convolutional layer and gradually added more layers. The optimal number of convolutional layers is determined when adding more layers does not significantly increase the AUC values. The relationship between the AUC values computed on the testing dataset and the depth of the CNNs was quantified and shown in Fig. 10. The AUC values were not significantly increased after six convolutional layers were included. Therefore, the CNN architecture that possesses six convolutional layers was selected for this example.

## Data Availability

Code and data will be made publicly available upon acceptance of the paper.
